# Prune belly syndrome in a neonate with severe bilateral hydronephrosis: A rare case report from Somalia

**DOI:** 10.1016/j.ijscr.2024.110310

**Published:** 2024-09-18

**Authors:** Abdirahman Omer Ali, Abdirahman Ibrahim Said, Mohamed Ahmed Abdilahi, Abdirahman Said Deheye, Abdisalam Hassan Muse

**Affiliations:** a College of Health Sciences, School of Medicine and Surgery, Amoud University, Borama, Somalia; b School of Postgraduate Studies and Research, Amoud University, Amoud Valley, Borama 25263, Somalia; c Al-Hayat Hospital, Pediatric Department, Somalia

**Keywords:** Prune belly syndrome, Cryptorchidism, Hydroureteronephrosis, Case report, Supportive care

## Abstract

**Introduction and importance:**

Prune belly syndrome (PBS), also known as Eagle-Barret syndrome, is a rare congenital disorder characterized by abdominal wall muscle underdevelopment, urinary system abnormalities, and cryptorchidism. This case report presents the clinical features, diagnosis, and management of PBS in a newborn. This is the first case report of prune belly syndrome in Somalia. The condition is estimated to occur in approximately 1 in 30,000 to 1 in 50,000 live births, making it a relatively uncommon presentation. Recognizing and managing this syndrome is crucial, as it can lead to significant morbidity and mortality if not addressed promptly.

**Case presentation:**

A term baby delivered without complications developed respiratory distress, jaundice, and urinary retention shortly after birth. Physical examination revealed abdominal distension, fluid in the abdomen, and bilateral undescended testes. Laboratory tests showed elevated bilirubin levels and abnormal blood counts. Ultrasound findings demonstrated bilateral hydroureteronephrosis and underdeveloped abdominal wall muscles. The limited resources and infrastructure in the healthcare setting in Somalia posed challenges in providing comprehensive care for this neonate.

**Clinical discussion:**

PBS is a rare congenital syndrome with a higher prevalence in males. Its exact cause is not fully understood, but genetic factors may play a role. The management of PBS in resource-limited settings can be particularly challenging. The differential diagnosis included sepsis, neonatal jaundice, and posterior urethral valves. The key interventions included supportive care, such as maintaining fluid and electrolyte balance, treating infections, and addressing any urinary tract abnormalities. The limited access to specialized pediatric urology services and advanced diagnostic tools, such as magnetic resonance imaging (MRI), hindered the ability to fully characterize the extent of the urinary tract abnormalities and plan definitive surgical interventions.

**Conclusion:**

Despite the constraints of the resource-limited setting, the supportive care and management strategies implemented led to an improvement in the baby's condition. This case highlights the importance of recognizing and managing Prune Belly Syndrome, even in environments with limited healthcare resources. Continued efforts to improve diagnostic capabilities and access to specialized care are crucial for optimizing the outcomes of patients with this rare and complex congenital disorder.

## Highlights

• A term neonate born in Somalia presented with respiratory distress, jaundice, and urinary retention shortly after birth.
• Key findings included bilateral hydronephrosis, underdeveloped abdominal wall muscles, and bilateral cryptorchidism, confirming the diagnosis of Prune Belly Syndrome (PBS).
• Diagnosis was supported by ultrasound imaging and clinical evaluation, highlighting the challenges of identifying PBS in resource-limited settings.
• The case underscores the importance of recognizing PBS early to prevent significant morbidity and mortality.
• Supportive care was the primary intervention, focusing on maintaining fluid and electrolyte balance, treating infections, and addressing urinary tract abnormalities.
• Financial constraints limited access to advanced diagnostic tools and surgical interventions, emphasizing the challenges faced in low-resource environments.
• The baby showed improvement by day 5, with successful breastfeeding and decreased jaundice.
• Initially required self-catheterization, but later began urinating normally as abdominal distension decreased.
• PBS is a rare congenital disorder with a higher prevalence in males and significant associated complications.
• The case highlights the need for a multidisciplinary approach for effective management, integrating multiple specialties to address the various aspects of the syndrome.
• The report emphasizes the importance of prompt recognition and management of PBS, even in resource-limited settings.
• Continued efforts to enhance diagnostic capabilities and access to specialized care are essential for improving outcomes in patients with PBS.
• Prune belly syndrome, cryptorchidism, hydroureteronephrosis, case report, supportive care

## 1 Introduction

Prune belly syndrome (PBS), also known as Eagle-Barret syndrome, triad syndrome, or Obrinsky syndrome, is a rare congenital disorder with an estimated incidence of 1 in 40,000 live births [1]. The condition is predominantly found in males, accounting for 95 % of cases [1]. PBS is characterized by a triad of symptoms: abdominal wall muscle underdevelopment. Urinary system abnormalities and cryptorchidism [2]. This syndrome is rarely reported in sub-Saharan Africa, with most paediatricians encountering very few cases in their clinical practice [3,4]. This case is written in line with the SCARE 2023 guideline [4].

## 2 Case presentation

A term baby with was delivered vaginally without complications to a 25-year-old gravida 3, para 2 mother. The baby's gestational age at birth a was 39 weeks by LMP with birth weight of 3.4 kg, and. The mother was not on any regular antenatal checkups (ANCs) with no prenatal ultrasound or laboratory investigations. There was no history of exposure to teratogenic drugs, radiation, or illness with fever and rashes during the pregnancy. Additionally, the mother had no known history of diabetes and there was no history of congenital anomalies in her family. The report lacks information about the presence of oligohydramnios during prenatal ultrasound imaging. Shortly after birth, the baby developed grunting, nasal flaring, tachypnea, and urinary retention. Family members reported no complications during prenatal care. On day 4, the baby developed severe jaundice extending to the feet and also family reported urinary retention.

## 3 Clinical findings and initial management

On examination, the child had severe jaundice all over the body with signs of respiratory distress; hypoxia, and tachypnea. He had a distended abdomen and fluid in the abdomen. He also had anuria and bilateral cryptorchidism. He did not have any obvious facial deformities and the rest of the physical examination were normal.

## 4 Diagnostic assessment

### 4.1 Laboratory tests

Laboratory tests revealed a total bilirubin level of 18.0, with direct bilirubin at 10.0. Complete blood count (CBC) showed a white blood cell count of 12.9, hemoglobin level of 17.8, and platelet count of 50.

### 4.2 Ultrasound findings

**Fig. 1:**
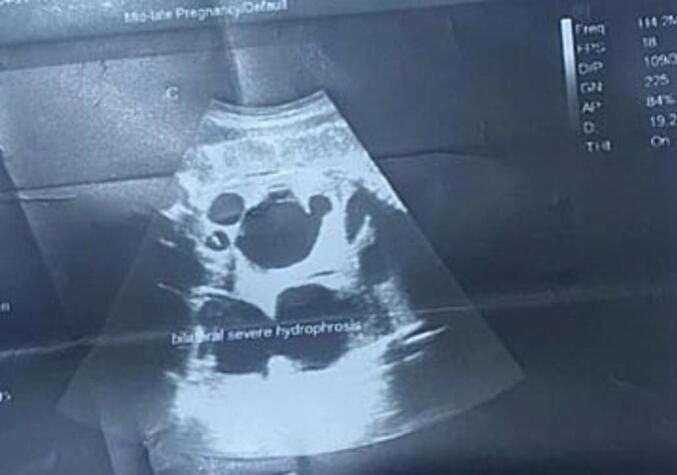
Both kidneys are significantly enlarged in size and have increased cortical echogenicity with thinned out cortical thickness. There is extensive pelvicalyceal and ureteric dilatation seen bilaterally.

**Fig. 2:**
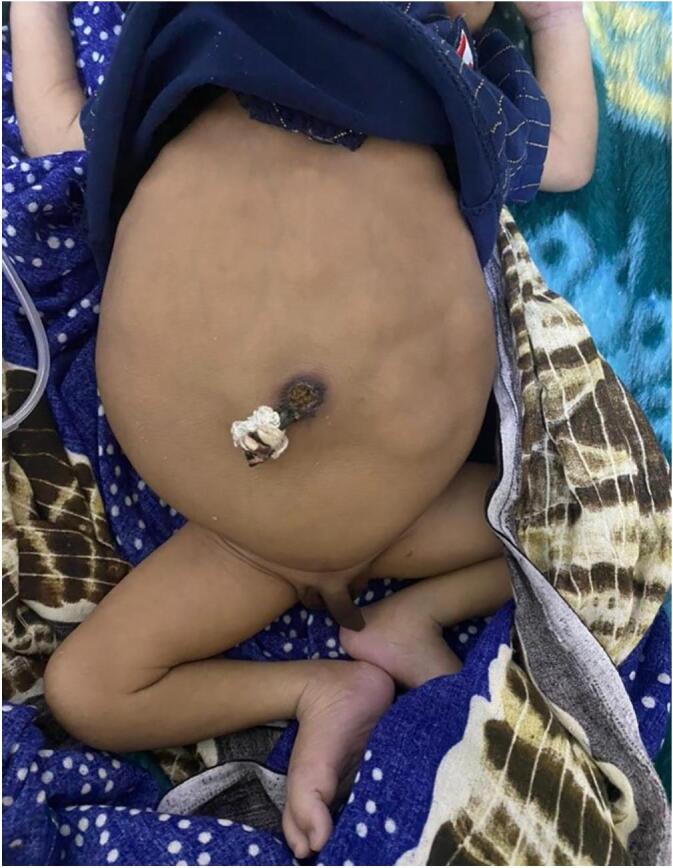
Prune Belly Syndrome-A newborn baby with abdominal distension, visible peristalsis, bilateral cryptorchidism and small penis, highlighting the characteristics features of this rare congenital disorder.

An ultrasound of the kidneys and urinary tract revealed bilateral hydroureteronephrosis, extensive pelvicalyceal and ureteric dilation bilaterally (Fig. 1). An abdominal ultrasound showed underdevelopment of the anterior abdominal wall with extremely thin muscular components, consistent with Prune Belly Syndrome (Fig. 2).

### 4.3 Voiding cystourethrography report

#### 4.3.1 Cystogram

The bladder is well distended and has a normal mucosal outline with no diverticula or filling defect. There is bilateral vesicoureteral reflux (VUR) with dilated tortuous ureters and bilateral abnormally lower abdominal location of kidneys with gross dilatation of the bilateral pelvicalyceal system.

#### 4.3.2 Impression

Bilateral grade-V VUR with severe hydroureteronephrosis with no sign of urethral obstruction.

### 4.4 Assessment and initial management

Having the classical triad of prune belly syndrome and features of sepsis, the final diagnosis of prune belly syndrome with early-onset neonatal sepsis was made. The baby was admitted to the neonatal intensive care unit (NICU) and connected to oxygen with continuous positive airway pressure (CPAP). Intravenous antibiotics (ampicillin and cefotaxime) were initiated, and intense phototherapy was started. A blood transfusion of one unit was given due to low platelet count (50,000). In addition, the urine was intermittently drained with 6 Fr straight catheter.

### 4.5 Interventions

Due to limited financial resources, the originally planned surgical management, which involved sequential interventions including orchidopexy, urinary tract reconstruction, and abdominoplasty, could not be carried out within the required timeframe.

### 4.6 Outcomes and follow-up

The patient's condition improved on day 5, with good breastfeeding and a reduction in jaundice and bilirubin levels. Initially, the baby required self-catheterization, but later on, he started urinating well, and abdominal distension decreased. The report lacks detail on any interim management strategies or adjustments made due to these limitations. Including information on the follow-up care plan and any future interventions would give a more complete understanding of the patient's progress and management.

## 5 Discussion

Prune belly syndrome (PBS) is a rare congenital disease characterized by the involvement of the abdominal wall muscles, urological system, and the occurrence of bilateral cryptorchidism (undescended testicles). It is estimated to occur in approximately 1 in 30,000 to 40,000 live births, with a higher prevalence in males [5]. The perinatal mortality rate in PBS ranges from 10 % to 25 % and is closely associated with the severity of pulmonary hypoplasia resulting from oligohydramnios caused by renal dysplasia and urinary tract abnormalities, leading to Potter sequence [6].

The classification of PBS is based on antenatal and postnatal features. Category 1 includes patients with severe renal dysfunction and pulmonary hypoplasia, which leads to a high mortality rate. Category 2 patients exhibit the classic triad of features with varying degrees of renal dysplasia, and some may require early dialysis. Category 3 patients have normal renal function and milder phenotypic features of PBS [5].

The exact cause of PBS is not fully understood. While it often appears sporadically, there have been familial cases and associations with chromosomal anomalies, such as trisomy 21 and large deletions in the long arm of chromosome 6. It has been suggested that PBS may have an autosomal recessive mode of inheritance, including a mutated variant of the CHRM3 gene on chromosome 1q43 [5].

A study conducted in Sudan focused on PBS and found that ultrasound examinations revealed various combinations of urinary tract anomalies, with hydronephrosis and hydroureter being the most common. The study's results indicated that a significant proportion of PBS patients developed chronic kidney disease (53.3 %) during the last follow-up, while only a small percentage retained normal kidney function (13.4 %). Hydronephrosis, a major renal anomaly in PBS, plays a critical role in the progression towards chronic kidney disease. A case study revealed severe hydronephrosis in a patient with PBS, although renal function tests remained within normal limits. However, ongoing monitoring is necessary due to the increased risk of chronic kidney disease associated with PBS [7].

The diagnosis of PBS can occur prenatally or shortly after birth. Prenatal ultrasonography, typically performed in the second trimester, can often identify characteristic features of PBS, such as hydroureteronephrosis and megacystis. After birth, the characteristic appearance of the abdominal wall aids in the diagnosis. Radiography, renal ultrasound, and voiding cystourethrography are used for confirmation and further evaluation [3,6,9]. Another study emphasized the importance of bilateral hydroureter and hydronephrosis, a distended, thin-walled bladder, and oligohydramnios as fundamental findings in the sonographic diagnosis of PBS [8]. The presence of pulmonary hypoplasia and severe renal dysfunction in PBS is associated with a high mortality rate of up to 100 % within the first few days of postnatal life [9]. In the case discussed, PBS was clinically diagnosed in the delivery room and later confirmed with postnatal ultrasonography. A comprehensive clinical assessment, radiography, and ultrasound results are crucial for a definitive diagnosis of PBS [1].

Due to the rarity and wide range of severity of PBS, there are currently no established guidelines or consensus regarding its management. The lack of standardized treatment is further complicated by the diverse nature of the syndrome. However, certain considerations should be taken into account. These include addressing the repair of the abdominal wall flaccidity, managing various urinary tract anomalies, and correcting cryptorchidism as a necessary step. The timing and staging of surgical interventions are also factors that need to be considered [5,7].

In cases where PBS is diagnosed prenatally through intrauterine ultrasonography, therapeutic options, such as in utero placement of a vesicouterine shunt, can be considered to prevent renal damage, which may impact the prognosis later on [9,10]. Treatment of PBS involves surgical correction of the abdominal wall defect and urinary abnormalities, early orchiopexy, and supportive management of associated defects [8].

The optimal care for PBS requires a multidisciplinary team approach. Pediatric urologists lead the team, while physical therapists, pulmonologists, nephrologists, gastroenterologists, dieticians, and other specialists provide support. The team's goals include promoting growth, preparing for potential surgeries, stabilizing renal function, addressing pulmonary issues, improving intestinal motility, and addressing orthopedic and psychiatric needs in older children [8].

## 6 Conclusion

Prune belly syndrome (PBS) is a rare congenital disorder characterized by abdominal wall muscle underdevelopment, urinary system abnormalities, and cryptorchidism. Prompt recognition, accurate diagnosis, and multidisciplinary care are crucial for managing PBS effectively. Financial constraints may impact the timing of surgical interventions, emphasizing the need for creative solutions. Supportive measures, such as oxygen therapy and antibiotics, play a vital role in improving outcomes. Long-term monitoring is necessary to assess renal function and detect potential complications. Further research is needed to establish standardized guidelines for the diagnosis and management of PBS.

## Patient consent

Written informed consent was obtained from the patient and the patient's parents for publication and any accompanying images. A copy of the written consent is available for review by the Editor-in-Chief of this journal on request.

## Ethical approval

The study protocol, case investigation, and consent form were thoroughly examined by the institutional review board of the College of Health Sciences at Amoud University. They granted approval for the study, along with the Ministry of Health and Borama Regional Hospital in Awdal Region, Somaliland (BRH-80/2024). Prior to participation, written informed consent was obtained from every individual involved.

## Funding

Not applicable.

## Author contribution

Dr abdirahman omer ali, Dr Abdirahman Ibrahim Said, Dr. Mohamed Ahmed Abdilahi, and Dr. Abdirahman Said Daheye these individuals contributed to taking history and providing care to the patient throughout her hospital stay. Additionally, Dr. Abdisalam Hasan Muse, Dr Abdirahman Ibrahim Said and Dr Abdirahman Omer Ali contributed to the development of the manuscript.

## Guarantor

Dr. Abdirahman Omer Ali and Dr. Abdirahman Ibrahim Sicid is guarantor of this paper.

## Conflict of interest statement

The authors affirm that there are no conflicts of interest pertaining to the publication of this article.
